# Genetic Variation and Phylogenetic Analysis of Indonesian Sugarcane (*Saccharum officinarum* L.) based on Internal Transcribed Spacer (ITS-nrDNA)

**DOI:** 10.21315/tlsr2026.37.1.3

**Published:** 2026-03-31

**Authors:** Ganies Riza Aristya, Thoriq Abdul Halim, Tiara Putria Judith, Rina Sri Kasiamdari, Janis Damaiyani, Heri Prabowo

**Affiliations:** 1 Laboratory of Genetics and Breeding, Department of Tropical Biology, Faculty of Biology, Universitas Gadjah Mada, Yogyakarta 55281, Indonesia; 2 Laboratory of Plant Systematics, Department of Tropical Biology, Faculty of Biology, Universitas Gadjah Mada, Yogyakarta 55281, Indonesia; 3 Directorate of Scientific Collection Management, National Research and Innovation Agency, Cibinong, West Java 16911, Indonesia; 4 Indonesian Agency for Agricultural Development and Modernization, Malang, East Java 65152, Indonesia

**Keywords:** DNA Barcoding, ITS, Phylogenetic Tree, *Saccharum officinarum*

## Abstract

Sugarcane (*Saccharum officinarum* L.) is a crucial agricultural crop in global sugar production. Over the past decade (2010–2019), sugarcane production in Indonesia has experienced annual fluctuations, reaching its peak in 2013 with 35.5 million tons and declining to 27.7 million tons in 2019. Concurrently, Indonesia’s sugar production only met 38% of domestic demand, necessitating the development of superior sugarcane cultivars with high sucrose content and resistance to pests and diseases. The aim of this study was to identify and determine the relationship among three sugarcane cultivars in Indonesia using DNA barcoding and constructing a phylogenetic tree based on ITS nuclear ribosomal DNA sequences. The ITS region was amplified using the primers ITS1 and ITS4, yielding a final base pair length of 531 bp. The sequences were then analysed to construct a phylogenetic tree using Maximum-Likelihood (ML) and Bayesian Inference (BI) methods. Sequence analysis was also conducted to determine genetic distances, polymorphic sites, haplotype distributions, and Principal Coordinate Analysis (PCoA). Phylogenetic tree construction grouped all cultivars into three distinct clades for both methods, with all three cultivars placed in the same clade with a bootstrap value of 94 for ML and a posterior probability of 1 for BI. Haplotype distribution showed that cultivars POJ and Pringu belonged to the same group, and PCoA analysis indicated no separation based on geographic origin, with some compositions similar to those of other countries such as Mexico, Taiwan and China.

**HIGHLIGHTS**

- ITS region successfully amplified, resulting in a base length of 531 bp including ITS1 and ITS2 regions.
- The phylogenetic tree divided all sugarcane samples into three clades, with all three Indonesian samples falling into the same clade.
- The haplotype values indicate that three sugarcane cultivars in Indonesia (Kidang Kencana, POJ and Pringu) have the same composition as several other countries.

## INTRODUCTION

Sugarcane (*Saccharum* spp.) is a widely cultivated agricultural crop due to its superior growth compared to most other plants, attributed to its high efficiency in photosynthesis as a C4 plant. Sugarcane plays a vital role in global sugar production, contributing to about 70%, and holds potential as a biomass source for biofuel (Dinesh Babu *et al*. 2022). Global sugar production reached 1,907,024,730 tons in 2018, with Brazil and India being the largest producers (FAO 2021). In Indonesia, Java Island accounts for approximately 61% of the total national sugarcane production, with 64% originating from East Java (Widyasari *et al*. 2022). Sugar production in Indonesia reached 2.4 million tons in 2022, increasing by 2.31% compared to the previous year (Badan Pusat Statistik 2023).

Current commercial sugarcane varieties are the result of traditional crosses between *Saccharum officinarum* and wild *Saccharum spontaneum* varieties. The majority of the genome is derived from *S. officinarum* (80%), followed by *S. spontaneum* (10%–15%), with a small portion being chromosome recombinations (5%–10%) (Garsmeur *et al*. 2018). The increasing demand for sugar has prompted farmers to exploit sugarcane’s genetic diversity to the fullest extent possible. The varietal selection process in sugarcane domestication has led to a narrowing of genetic diversity, creating significant challenges in increasing sugarcane yields, including polyploid genomes hindering flowering, low fertility and long breeding cycles (10 years–15 years). Hence, an integrated approach between conventional and molecular breeding techniques is needed (Dinesh Babu *et al*. 2022).

Over the past decade (2010–2019), sugarcane production in Indonesia has experienced annual fluctuations, reaching its peak in 2013 with 35.5 million tons. However, in 2019, sugarcane production declined to 27.7 million tons. Over the five years preceding 2019, sugarcane production continued to decline. This decrease was caused by a reduced interest among farmers in cultivating sugarcane due to decreasing profits. The main contributing factors were increased production costs and stagnant sugarcane prices, which did not align with the rising production costs. Currently, sugar production only meets 38% of the total demand in Indonesia, with the remainder being imported (Widyasari *et al*. 2022).

Sugarcane identification is a crucial step in agricultural activities, including breeding, seed production and trade, and inspection (Ali *et al*. 2021). Although identification based on morphological and agronomic traits has been commonly used in sugarcane classification, errors can occur due to environmental variations that affect plant morphology, resulting in changes that do not represent the true genetic diversity. Therefore, molecular biology techniques such as DNA barcoding have become reliable options for plant identification, including sugarcane, especially in the breeding stage (Medeiros *et al*. 2020). Currently, Internal Transcribed Spacer (ITS) sequences have become popular in research for identifying and determining relationships among plants. Research conducted by Michel *et al*. (2016), using ITS2 sequences as barcodes in DNA barcoding of herbal plants in China, showed that ITS2 could be an effective alternative to replace *matK* and *rbcL*, especially since some herbal plants lose their chloroplasts. Furthermore, a study by Yang *et al*. (2016) used Single Nucleotide Polymorphism (SNP) from ITS sequences on nuclear ribosomal DNA (nrDNA) to identify *Saccharum* species. This helped improve understanding of sequence order in current sugarcane cultivars. The objective of this study was to identify and determine the relationship among three sugarcane cultivars in Indonesia using DNA barcoding and constructing a phylogenetic tree based on ITS-nrDNA sequences.

## MATERIALS AND METHODS

### Plant Materials

**FIGURE 1:**
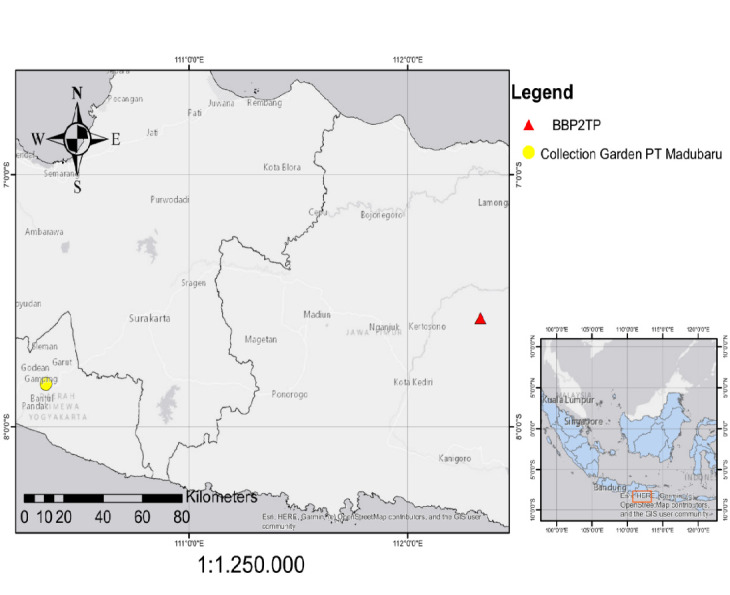
Sampling locations map. (Source: Google Earth)

Three sugarcane specimens of the Proefstation Oost-Java (POJ), Kidang Kencana (KK) and Pringu cultivars were obtained from the PT Madubaru collection garden in Bantul, Yogyakarta Special Region, and East Java detailed in Table 1 and Fig. 1. The samples used were the third to fifth leaves from the sugarcane plant’s apex (Aristya *et al*. 2020). Leaves were collected by cutting the base of each leaf with scissors, dividing them into 3 to 4 pieces, and placing them in an icebox to maintain a cold temperature. The samples were stored in the Genetics and Breeding Laboratory at the Faculty of Biology, Universitas Gadjah Mada (UGM), in a freezer at –20°C for further analysis. To broaden the analysis, we included a total of 17 ITS sequence samples from various collections worldwide, including Mexico, Taiwan, India, China, Saudi Arabia and the US. These additional samples were included to enhance our understanding of the genetic variation and relationships among sugarcane cultivars worldwide.

### DNA Extraction

The total of 100 g of sugarcane young leaves were collected and powdered after freezing in liquid nitrogen with the leaf veins removed. The isolation was performed using the Geneaid DNA Kit (Geneaid Biotech Ltd., Taiwan) following the manufacturer’s procedure.

### ITS Sequence Amplification by PCR Method

Amplification of the nrDNA ITS region was performed using the T100 Thermal-Cycler PCR machine (Bio-Rad Laboratories Inc., USA) with two universal primers for the ITS region, ITS1 (5′-TCCGTAGGTGAACCTGCGG-3′) and ITS4 (5′-TCCTCCGCTTATTGATATGC-3′) (Yang *et al*. 2018). The PCR machine was run with a pre-denaturation protocol at 95°C for 5 min, followed by 35 cycles of denaturation at 95°C for 30 sec, annealing at 54°C for 30 sec and extension at 72°C for 45 sec, followed by a final extension at 72°C for 5 min. The PCR products were electrophoresed on a 1% agarose gel and run at 100V for 30 min containing 1× TBE buffer.

### Sequencing ITS

Sequencing was carried out using an Applied Biosystems 3500 Genetic Analyzer (Applied Biosystems, USA). DNA was purified using Exosap-IT and incubated at 37°C for 15 min and 80°C for 15 min using the BioRad T100 PCR machine. Subsequently, sequencing was performed with a 10 μL volume for each reaction, consisting of 1 μL of DNA template, 0.5 μL of each forward and reverse primer (3.2 pM/μL), 1 μL of Big Dye Terminator, 2 μL of 5× buffer and 6 μL of nuclease-free water (NFW). The sequencing cycle was run with an initial pre-denaturation protocol at 96°C for 1 min, followed by denaturation at 96°C for 10 sec, annealing at 50°C for 5 sec, and extension at 60°C for 4 min, repeated for 25 cycles. Sequencing products were purified using the Bigdye X-Terminator Purification kit, and the results were collected using the ABI 3500 Genetic Analyzer machine. Subsequently, sequencing data analysis was performed using the SeqA software.

### Editing and Alignment of ITS Sequences

Ambiguous DNA nucleotides were manually edited using GeneStudio software. To validate the sugarcane species, consensus results were compared with data available on GenBank using the BLAST program (https://blast.ncbi.nlm.nih.gov/Blast.cgi). In this study, we aligned a total of 17 sugarcane ITS sequences, resulting in a fragment length of 531 bp using MEGA11 software’s align by MUSCLE menu. These sequences were used for subsequent intraspecific analysis. For phylogenetic analysis, two samples were added as outgroups, *Miscanthus sinensis* (AB761569) and *Tripidium arundinaceum* (MN813475) during the alignment process without affecting the previously mentioned fragment length of 531 bp.

### Phylogenetic Analysis

For phylogenetic tree reconstruction, we analysed DNA sequences using the best scheme analysis using the PartitionFinder 2 software based on Python coding. Subsequently, the alignment results were processed using the Nexus program to run the Maximum-Likelihood (ML) and Bayesian Inference (BI) programs. Phylogenetic trees were constructed using ML analysis with IQ-Tree version 1.6.10 software with 1,000 bootstrap replicates (Nguyen *et al*. 2015) with HKY+G: Subset1 substitution model based on Partition Finder analysis. BI analysis was performed using MrBayes version 3.2.6 software (Ronquist *et al*. 2012) statistically for each node of the phylogenetic tree based on Markov Chain Monte Carlo (MCMC) and repeated for 4,000,000 generations using HKY model substitution.

### Genetic Variation Analysis, Haplotype Network and Principal Coordinate Analysis

Intraspecific genetic variation analysis in this study included haplotype number (h), haplotype diversity (Hd), the number of polymorphic sites and the number of parsimony sites analysed using DnaSP software (Rozas *et al*. 2017). Furthermore, Haplotype Network was analysed using PopART version 1.7 software, and Principal Coordinate Analysis (PCoA) was analysed using GenAIEx 6 software (Peakall & Smouse 2012).

## RESULTS

### PCR Amplification Results

**FIGURE 2:**
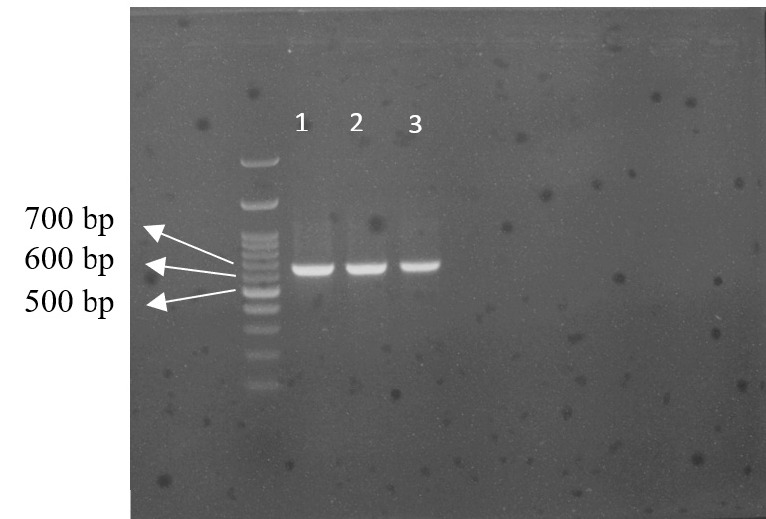
Electropherogram 3 of three sugarcane cultivars. *Note*: M = Marker 100 bp; 1: POJ (Proefstation Oost Java); 2: KK (Kidang Kencana); 3: Pringu.

The results indicate that the amplification of the ITS nrDNA sequence produced a DNA fragment approximately 678 bp in length shown in Fig. 2. The amplification was carried out using specific primers ITS1 and ITS4 (Besse 2014), which can amplify the entire ITS region (ITS1, 5.8S, ITS2).

### Sequencing Results

All sequences that were free from ambiguity were further analysed using the BLAST program on NCBI. Homology of all sugarcane sequence samples with other sequences in the database showed values > 98% (Table 2), indicating that the samples contained the ITS region in nrDNA and were conserved.

#### Phylogenetic tree construction and genetic distance

**FIGURE 3:**
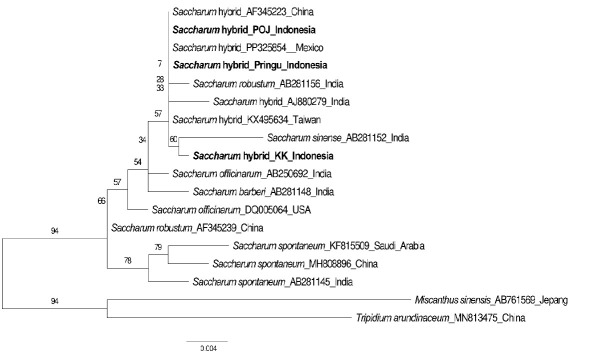
The results of constructing a Maximum-Likelihood phylogenetic tree using the Kimura-2-parameter model with 1,000× bootstrap support for *Saccharum* spp. and outgroup from GenBank based on ITS sequences (531 bp). The numbers on the branches of the tree indicate bootstrap values.

**FIGURE 4:**
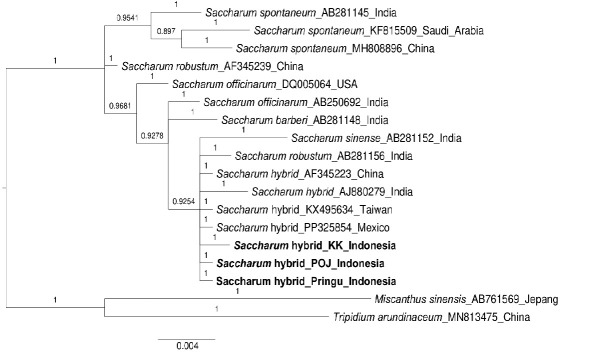
BI phylogenetic tree of *Saccharum* spp. and outgroup from GenBank based on ITS sequences (531 bp). The numbers on the branches of the tree indicate bootstrap values.

In this study, a total of 18 sequences were analysed, with additional samples from GenBank as outgroups, namely *Miscanthus sinensis* (AB761569) and *Tripidium arundinaceum* (MN813475). The results of the ML and BI phylogenetic trees showed differences in the position of the KK cultivar from Indonesia. Additionally, the tree analysis results showed good bootstrap support in the BI tree (Fig. 3) and low bootstrap support in the ML tree (Fig. 4).

Fig. 4 illustrates the results of constructing a phylogenetic tree using the Maximum-Likelihood method with the Kimura-2-parameter model and 1,000× bootstrap support. The horizontal line lengths on the tree signify the relationship between species, where longer lines indicate more distant relationships (Liu *et al*. 2017). In the ML tree, all cultivars are divided into 3 clades, with all three samples from Indonesia falling into the same clade. This result contradicts statements by Irvine (1999) and Yang *et al*. (2016), which suggest that the division of clades in sugarcane should only be into 2, namely *S. spontaneum* and *S. officinarum*. The same result is also shown by the phylogenetic tree using the BI method with a posterior probabilities value of 1 shown in Fig. 3. Cultivar AF345239, which is *S. robustum*, is separated into a different clade from *S. officinarum*. The separation of *S. robustum* (AF345239) is likely due to mismatches and base transversions that occur in this cultivar’s sequences (Table 3), which have a greater weight in clustering influence compared to the indels that occur in *S. robustum* (AB281156) (McDonald *et al*. 2011).

Genetic distances were calculated based on pairwise distances using the Kimura 2-parameter method among all sequence data, yielding varied results ranging from 0.19 to 2.12 (Table 4). The smallest genetic distance of 0.19 was found between POJ and KK, while the largest genetic distance of 2.12 was observed between AB281152 and KF815509. The average genetic distance measure at 0.84, indicating considerable genetic variation and genetic divergence among the 16 sequences based on the ITS region.

### Genetic Variation

Based on Table 3, there are polymorphic sites consisting of 12 Single Nucleotide Polymorphism (SNP) and 12 parsimony sites. The ITS sequences show abundant variations such as deletions, base transversions G–A and C–T, and base mismatches C–G and T–A. Tables 5 and 6 show the results and details of haplotypes formed based on ITS sequences in all samples, dividing the 16 samples into 12 groups. Samples POJ and Pringu fall into the same group in H6, while KK falls into group H11. Haplotypes are formed based on the detailed polymorphism range at specific loci among species, so if different species fall into different groups, it can be said that there are differences in allele combinations at those loci (De Mendonça Vilela *et al*. 2017). The differences shown by these samples may occur because sugarcane is a polyploid plant resulting from the crossing of *S. officinarum* and *S. spontaneum* (Piperidis *et al*. 2010), so in the process of searching for superior cultivars, there have been many changes in allele arrangements due to differences in combinations inherited from parental alleles (De Mendonça Vilela *et al*. 2017).

### Haplotype Network and Principal Coordinate of Analysis

**FIGURE 5:**
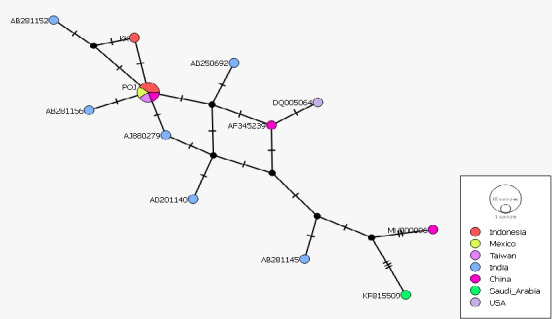
Haplotype network of 16 *Saccharum* cultivars based on gen barcode ITS. Circle size indicates the number of samples in the haplotype network analysis. The lines connecting haplotypes indicate the evolutionary route between haplotypes, and the short lines indicate mutation points between haplotypes. Each colour indicates the origin of the sample.

**FIGURE 6:**
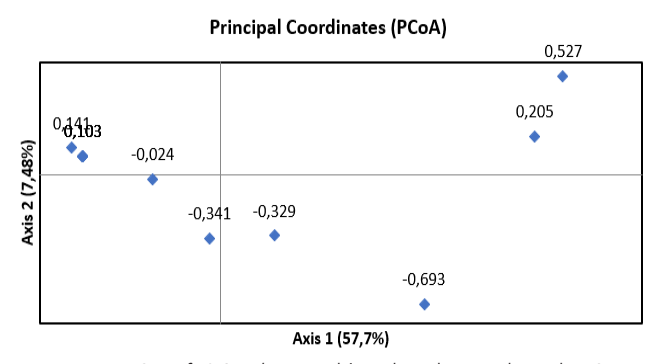
PCoA of 16 *Saccharum* cultivars based on gen barcode ITS.

The haplotype network analysis (Fig. 5) and principal coordinate of analysis (PCoA) (Fig. 6) indicate that there is no geographic separation or have the same composition as some other countries such as Mexico, Taiwan and China. These results are consistent with the phylogenetic tree construction in this study. This similarity is attributed to the colonial Dutch nobilisation, which resulted in the “noble cane” produced from the crossing of the Black Cheribon cultivar (as *S. officinarum*) with the Kassoer cultivar (as *S. spontaneum*). Current sugarcane cultivars are hybrid clones produced from *S. officinarum* and *S. spontaneum* with several genes incorporated from *S. barberi* and *S. sinense* (Dinesh Babu *et al*. 2022).

## DISCUSSION

In the results of this study, ITS sequences can show the relationship of sugarcane relationship to the intraspecific level because they contain more informative sites as shown in Table 5, so that these sequences are often used in identification and knowing the relationship of plants (Yang *et al*. 2016; Michel *et al*. 2016). The many site variations of ITS shown in Table 3 affect the clade formation of the 16 species used in this study and make ITS-nrDNA can be used as a good source for studying sugarcane relationships. The 16 sequences clustered into 12 haplotypes (Table 6). The haplotype network shown the same results as the phylogenetic tree in the form of no separation based on geography or had the same composition as several other countries, including Mexico, Taiwan and China. This is possible because it is related to the nobilisation carried out by the Dutch colony (Dinesh Babu *et al*. 2022) and currently existing cultivars are the result of hybrid clones derived from *S. officinarum* and *S. spontaneum* with several genes incorporated from *S. barberi* and *S. sinense* (Dinesh Babu *et al*. 2022) and the introduction of broodstock by between countries also affects the relationship of all current sugarcane cultivars (Widyasari *et al*. 2022).

## CONCLUSION

Based on the results of this study, we conclude that the ITS nrDNA sequences of the three sugarcane cultivars were successfully amplified, resulting in a final base length of 531 bp. Phylogenetic analysis using ML and BI divided all 16 sugarcane samples into three clades, with all three Indonesian samples falling into the same clade. The haplotype values indicate that the POJ and Pringu cultivars belong to the same haplotype group (H6), while KK belongs to group H11. Haplotype network analysis and PCoA based on ITS sequences show that the three sugarcane cultivars in Indonesia have the same composition as several other countries.

## Figures and Tables

**TABLE 1:** List sample sugarcane cultivar collections.

Cultivars	Collection time	Location sites	Latitude	Logitude
POJ	14 November 2023	PT. Madubaru, Bantul, Daerah Istimewa Yogyakarta	−7.830904116563534	110.34557465823727
KK			−7.830904116563534	110.34557465823727
Pringu	2018	Center for Plant Breeding and Plant Protection Surabaya	−7.53884361048272	112.32242651245463

**TABLE 2:** BLAST result.

Sample/Accession number	Description	Percent similarity (%)
POJ	*S*. hybrid	100
Pringu	*S*. hybrid	100
KK	*S*. hybrid	99.81
PP325854	*S*. hybrid	100
KX495634	*S*. hybrid	100
AJ880279	*S*. hybrid	100
AB250692	*S. officinarum*	100
AB281148	*S. barberi*	100
AB281156	*S. robustum*	100
AB281152	*S. sinense*	100
AB281145	*S. spontaneum*	100
AF345223	*S*. hybrid	100
AF345239	*S. robustum*	100
MH808896	S. spontaneum	100
KF815509	*S*. hybrid	100
DQ005064	*S. officinarum*	100

**TABLE 3:** Polymorphic sites of 16 sugarcane samples based on ITS region.

Nucleotide site number	3	10	11	17	27	28	36	37	39	43	62	68	69	70	96	104	126	137	168	171	324	372	451	509
POJ	C	G	C	C	G	C	C	T	C	C	C	C	C	C	C	C	A	G	C	C	C	-	G	C
Pringu	·	·	·	·	·	·	·	·	·	·	·	·	·	·	·	·	·	·	·	·	·	·	·	·
KK	·	·	·	T	·	·	·	·	·	·	·	·	·	·	·	·	·	·	·	·	·	·	·	·
PP325854	·	·	·	·	·	·	·	·	·	·	·	·	·	·	·	·	·	·	·	·	·	·	·	·
KX495634	·	·	·	·	·	·	·	·	·	·	·	·	·	·	·	·	·	·	·	·	·	·	·	·
AJ880279	·	·	·	·	·	T	·	·	·	·	·	·	·	·	·	·	·	·	·	·	·	·	·	·
AB250692	·	·	·	·	·	·	·	·	·	·	·	·	·	·	·	G	T	·	·	·	·	·	·	·
AB281148	·	·	·	·	·	·	·	·	·	T	·	·	·	·	A	·	T	·	·	·	·	·	·	·
AB281156	-	·	-	·	·	·	-	-	T	·	-	·	·	·	·	·	·	·	·	·	·	·	·	·
AB281152	A	C	G	G	·	-	-	·	·	·	·	·	·	·	·	·	·	·	·	·	·	·	·	·
AB281145	A	·	T	·	·	·	·	·	·	T	·	·	·	T	·	·	T	·	·	T	T	·	·	·
AF345223	·	·	·	·	·	·	·	·	·	·	·	·	·	·	·	·	·	·	·	·	·	·	·	·
AF345239	A	·	·	·	·	·	·	·	·	·	·	·	·	·	·	·	T	·	·	·	T	·	·	·
MH808896	A	·	·	·	·	·	·	·	·	T	·	G	·	T	·	·	T	·	·	·	T	C	A	T
KF815509	A	·	·	·	T	·	·	·	·	T	·	·	T	T	·	·	T	·	T	·	T	C	A	·
DQ005064	·	·	·	·	·	·	·	·	·	·	·	·	·	·	·	·	T	A	·	·	T	·	·	·

**TABLE 4:** Genetic distance of 16 *Saccharum* cultivars based on gen barcode ITS (%).

	1	2	3	4	5	6	7	8	9	10	11	12	13	14	15	16
1																
2	***															
3	0.19	0.19														
4	***	***	0.19													
5	***	***	0.19	***												
6	0.38	0.38	0.57	0.38	0.38											
7	0.38	0.38	0.57	0.38	0.38	0,76										
8	0.57	0.57	0.76	0.57	0.57	0.57	0.57									
9	0.19	0.19	0.38	0.19	0.19	0.58	0.57	0.77								
10	0.76	0.76	0.76	0.76	0.76	0.95	1.15	1,34	0.58							
11	1.34	1.34	1.53	1.34	1.34	1.34	1.34	1.14	1.16	1.53						
12	***	***	0.19	***	***	0.38	0.38	0.57	0.19	0.76	1.34					
13	0.57	0.57	0.76	0.57	0.57	0.95	0.57	0.76	0.57	0.95	0.76	0.57				
14	1.53	1.53	1.72	1.53	1.53	1.53	1.53	1.34	1.55	1.92	0.95	1.53	0.95			
15	1.72	1.72	1.92	1.72	1.72	1.72	1.72	1.53	1.74	2.12	1.15	1.72	1.15	0.95		
16	0.57	0.57	0.76	0.57	0.57	0.95	0.57	0.76	0.77	1.34	1.15	0.57	0.38	1.34	1.53	

*Notes:* 1 = POJ; 2 = Pringu; 3 = KK; 4 = PP325854; 5 = KX495634; 6 = AJ880279; 7 = AB250692; 8 = AB281148; 9 = AB281156; 10 = AB281152; 11 = AB281145; 12 = AF345223; 13 = AF345239; 14 = MH808896; 15 = KF815509; 16 = DQ005064. The notation *** signifies a genetic distance of 0, indicating no genetic distance between those samples.

**TABLE 5:** Genetic variation of *Saccharum* sample isolates and references from NCBI.

Number of samples	Number of haplotypes	Haplotype diversity	Nucleotide diversity
16	12	0.917 ± 0.064	0.00692

**TABLE 6:** Haplogroup of 16 *Saccharum* cultivars based on gen barcode ITS.

Haplotype	No. of samples	Sample code
H1	1	AB281152
H2	1	AF345239
H3	1	MH808896
H4	1	KF815509
H5	1	AB281145
H6	5	**POJ; Pringu;** PP325854; KX495634; AF345223
H7	1	DQ005064
H8	1	AB250692
H9	1	AB281148
H10	1	AJ880279
H11	1	**KK**
H12	1	AB281156
